# Potential Cytoprotective Activity of Ozone Therapy in SARS-CoV-2/COVID-19

**DOI:** 10.3390/antiox9050389

**Published:** 2020-05-06

**Authors:** Gregorio Martínez-Sánchez, Adriana Schwartz, Vincenzo Di Donna

**Affiliations:** 1 Scientific Advisor, Freelance, 60126 Ancona, Italy; 2 Clinica Fiorela Madrid, 28035 Madrid, Spain; adriana@clinicafiorela.com; 3 Dama Salus Medical Center, 76125 Trani, Italy; drdidonnavincenzo@gmail.com

**Keywords:** ozone therapy 1, SARS-CoV-2, Keap1/Nrf2/ARE pathway, NF-κB, Nrf2

## Abstract

(1) Background: The emergence of severe acute respiratory syndrome coronavirus 2 (SARS-CoV-2) disease (COVID-19) in China at the end of 2019 has caused a large global outbreak. Systemic ozone therapy (OT) could be potentially useful in the clinical management of several complications secondary to SARS-CoV-2. The rationale and mechanism of action has already been proven clinically in other viral infections and has been shown in research studies to be highly effective at decreasing organ damage mediated by inflammation and oxidative stress. This review summarizes the OT studies that illustrate the possible cytoprotective mechanism of action of ozone and its physiological by-products in target organs affected by SARS-CoV-2. (2) Methods: This review encompasses a total of 74 peer-reviewed original articles. It is mainly focused on ozone as a modulator of the NF-κB/Nrf2 pathways and IL-6/IL-1β expression. (3) Results: In experimental models and the few existent clinical studies, homeostasis of the free radical and antioxidant balance by OT was associated with a modulation of NF-κB/Nrf2 balance and IL-6 and IL-1β expression. These molecular mechanisms support the cytoprotective effects of OT against tissue damage present in many inflammatory diseases, including viral infections. (4) Conclusions: The potential cytoprotective role of OT in the management of organ damage induced by COVID-19 merits further research. Controlled clinical trials are needed.

## 1. Introduction

Coronaviruses are important human and animal pathogens. At the end of 2019, a novel coronavirus was identified as the cause of a cluster of pneumonia cases in Wuhan (Hubei Province of China) and caused a large global outbreak representing a major public health issue [1]. It rapidly spread, resulting in an epidemic throughout China, with increasing cases reported globally. In February 2020, the World Health Organization designated the disease COVID-19, which stands for coronavirus disease 2019 [2]. The virus that causes COVID-19 is designated severe acute respiratory syndrome coronavirus 2 (SARS-CoV-2); previously, it was referred to as 2019-nCoV. SARS-CoV-2 is closely related to two bat-derived severe acute respiratory syndrome-like coronaviruses, bat-SL-CoVZC45 and bat-SL-CoVZXC21, in particular BetaCoV/bat/Yunnan/RaTG13/2013 are similar to the human SARS-CoV-2 [3]. It is shown to have large genetic diversity and rapid evolution [4].

SARS-CoV-2 is spread by human-to-human transmission via respiratory droplets or direct contact, and infection has been estimated to have a mean incubation period of 6.4 days and a basic reproduction number of (2.24–3.58) days [1]. Among patients with pneumonia caused by SARS-CoV-2, fever was the most common symptom, followed by cough, malaise and dry cough at the prodromal phase [5]. Bilateral lung involvement with ground-glass opacity was the most common finding from computed tomography (CT) images of the chest. A progression of this radiographic phenomena was noted in CT images from the early stages of illness onset [6]. 

There are currently no antiviral drugs licensed by the U.S. Food and Drug Administration (FDA), by the European Medicines Agency (EMA), Spanish Agency of Medicines and Medical Devises (AEMPS) or by the Italian Drug Agency to treat patients with COVID-19. To the authors’ knowledge, no antiviral drugs to treat patients with COVID-19 have been licensed in any country in the world so far. This point has been officially confirmed by WHO: “Currently, there are no vaccines or specific pharmaceutical treatments available for COVID-19” [7]. Some in vitro or in vivo studies suggest potential therapeutic activity of compounds against related coronaviruses, but there are no available data from observational studies or randomized controlled trials in humans to support recommending any investigational therapeutics for patients with confirmed or suspected COVID-19 at this time. 

Remdesivir, an investigational antiviral drug, was reported to have in vitro activity against SARS-CoV-2 [8]. A small number of patients with COVID-19 have received intravenous Remdesivir for compassionate use outside of a clinical trial setting. A randomized placebo-controlled clinical trial of Remdesivir for treatment of hospitalized patients with pneumonia and COVID-19 has been implemented in China. The initial promising randomized open label trial of combination Lopinavir-Ritonavir finally did not show benefit [9]. Other drugs and treatment protocols that have been employed in Chinese clinical trials include Duranavir, Danoprevir, Cobisistat, Anti-CD147 Humanized Meplazumab, Eculizumab, Bevacizumab, Recombinant Human Angiotensin-converting Enzyme 2 (rhACE2), NK cells, Umbilical Cord (UC)-Derived Mesenchymal Stem Cells (MSCs), immunoglobulins, sphingosine-1-phosphate receptor regulators Fingolimod, hydroxy-chloroquine, intravenous vitamin C, Vitamin D, IFN beta, glucocorticoids, ozonated autohemotherapy (this is one of the many other compounds tried without successful available data yet; trial code: ChiCTR2000030165, ChiCTR2000030102 and ChiCTR2000030006) and traditional Chinese medicine remedies, but no results are available to date. Clinical trials of other potential therapeutics for COVID-19 are being planned [10,11]. No specific therapeutic drug has been found [12].

While the primary route of transmission appears to be through a respiratory route, SARS-CoV has been found in the intestinal tract, kidney and sweat glands of affected patients and thus may be excreted and transmitted via feces, urine and sweat. [13]. The angiotensin-converting enzyme 2 (ACE2) very likely serves as the binding site for SARS-CoV-2, the strain implicated in the current COVID-19 epidemic and similar to the strain SARS-CoV implicated in the 2002–2003 SARS epidemic [14]. In this epidemic, the major comorbidities in fatal cases included hypertension, diabetes, coronary heart disease, cerebral infarction, and chronic bronchitis. The source of the 2002–2003 SARS virus and its pathogenesis are still unconfirmed. 

Ozone (O_3_) is the triatomic allotrope form of oxygen, its oxidant potency is the third after fluorine and persulfate and it is higher than O_2_ [15]. Ozone therapy (OT), in the medical setting, employs a gas mixture of O_2_/O_3_, obtained from the modification of medical-grade oxygen using an ozone generator device that has to be administered in situ because of ozone’s short half-life (at 20 °C the O_3_ concentration is halved within 40 min, at 30 °C within 25 min) [15]. Usual clinical O_3_ concentrations range from 10 to 60 µg/mL (using O_2_ as vehicle) which, on a percentage basis, may range in a mixture of O_3_ (0.5–0.05%) and O_2_ (95–99.5%) [16]. The main mechanism of O_2_/O_3_ on human physiology fits the concept of oxidative preconditioning [17]. This concept has now been demonstrated at both the proteomic and genomic level [18], in in vitro studies and in clinical trials [19]. A calibrated oxidant stimulus by O_2_/O_3_ can modulate the endogenous antioxidant system and aid in the control of different pathological conditions [20]. The modulation of O_2_/O_3_ at the Keap1/Nrf2/ARE pathway and the reduction of IL-6 and IL-1β are involved in the mechanism of action of ozone [21]. This implies that the cytoprotective effect observed during the O_2_/O_3_ treatment may impact clinical conditions caused by SARS-CoV-2.

This review is focused on the cytoprotective effect of O_2_/O_3_ in different tissues, primarily though the modulation of the NF-κB/Nrf2 pathways and the IL-6 and IL-1β cytokines. There is preclinical and clinical evidence to support the potential role of OT in the prevention and management of cytotoxicity induced by different drugs and diseases including viral diseases [21,22,23,24]. The main mechanism is related to the modulation of the oxidative stress and pro-inflammatory cytokines. The Evidence Acquisition Terms included in the information search were: COVID-19, SARS-CoV-2, SARS, ozone, OT, viral pneumonia. Bibliographic databases consulted: MEDLINE/PubMed, SciELO, LILACS, PAHO, EMBASE, ZOTERO ISCO3, WHO International Clinical Trials Registry Platform and NIH. U.S. National Library of Medicine. The type of documents reviewed were published between 1980 to 2020, in Russian or English and included: Original articles, published thesis, clinical reports, ongoing clinical trials and bibliographic reviews. The exclusion criteria were the lack of free access to complete text due to financial constraints and/or, studies presenting inadequate scientific evidence. 

## 2. Potential Therapeutic Actions of Ozone in Viral Diseases 

Ozone can inactivate viruses via direct oxidation of its components [25]. However, the viricidal activity in vivo becomes uncertain when viruses are in biological fluids or, even worse, when they are intracellular (pneumocytes, hepatocytes, epithelia, CD4^+^ lymphocytes, monocytes, glial and neuronal cells) because the cell’s potent antioxidant system protects viral integrity [15]. That is why it is irrational to use direct I.V. injection of gas or other non-recommended methods of application of ozone [16,26]. OT represents a useful adjunctive and complementary therapy but neither ozone, nor H_2_O_2_ (one of the main O_3_ mediators) can reach sufficient concentrations in tissues because the plasma antioxidant capacity protects free pathogens and intracellular viruses are inaccessible [27]. In order to explore the efficacy of OT in viral diseases, Bocci and Paulesu [28] explained the possibility of how ozone may act in vivo. The following mechanisms may have some relevance:

1. A prolonged ozone therapeutic treatment appears able to induce an adaptation to oxidative stress, hence a re-equilibration of the cellular redox state, which is a fundamental process for inhibiting viral replication. The paradoxical mechanism by which ozone (a potent oxidant) can induce an antioxidant response, is currently demonstrated not only at a proteomic level, but also at a genomic one. Ozone applied at a therapeutic dose modulates the nuclear factor Nrf2 and NF-κB and induces the homeostasis of the antioxidant environment [18,29,30,31,32]. Oxidative stress and innate immunity have a key role in lung injury pathways that control the severity of acute lung cytotoxicity during viral infections like SARS [33].
2. The induction of antiviral cytokines such as IFN and the modulation of pro-inflammatory cytokines as IL-6, have been demonstrated by ozonating blood such as major autohemotherapy (MAH). Although ozone is a weak inducer, reinfused lymphocytes and monocytes during mayor autohemotherapy (MAH), can migrate through the lymphoid system, and activate other cells that, in time, will lead to the stimulation of the immune system [32,34]. This may represent an important process because it is known that an acute viral disease becomes more severe because the virus is particularly virulent, the heterogeneous viral population evolves rapidly and escapes immune control, or because the immune system becomes tolerant to viral antigens and becomes unable to counteract the infection. Moreover, besides the induction of HO-1 [18], a protective enzyme, there is also the release of some heat shock proteins (HSP) such as HSP60, HSP70 and HSP90 that also have an influence on viricidal activity. These proteins are potent activators of the innate immune system, and are able to induce the monocyte-macrophage system and the activation of antigen-presenting cells [15,35]. The evidence shows that, during the COVID-19 epidemic, the severe deterioration of some patients has been closely related to a dysregulated inflammatory process referred to as “the cytokine storm” [36,37].
3. Oxygen-ozone therapy improves oxygenation [38,39], especially in poorly oxygenated tissues [40]. Patients with SARS are prone to have mild non-specific hepatitis [41], lung fibrosis [42] and renal failure [43]. OT stabilizes hepatic metabolism and tend to normalize fibrinogen and prothrombin plasma levels in infected patients, suggesting an improvement of the hepatic protein synthesis [15]. There is extensive research demonstrating the cytoprotective effect of ozone to prevent oxidative damage to the heart [44,45], liver [46,47], lungs [48] and renal tissues [49]. The authors have described in a recent review, the cytoprotective effect of ozone to prevent and even to treat chemotherapy-induced damage in heart, liver, renal and lung tissue [22].
4. During blood ozonation ex vivo for the minor autohemotherapy, using ozone concentrations near 90 µg/mL per mL of blood, it may be feasible to induce the oxidation of free viral components, which could theoretically represent an inactivated and immunogenic vaccine [15,50,51].
5. Ozonized Saline Solution (O_3_SS). This method was formalized by the Ministry of Health of the Russian Federation in the early 1980s and has been officially implemented in public health hospitals, specifically for the specialties of orthopedics, dermatology, gynecology and obstetrics [16,52]. In 2004, it was also officially recognized in Ukraine. The beneficial effects of this therapy are supported by a large amount of scientific papers and strong clinical experience. [53]. The method consists of bubbling and saturating a physiological solution (0.9%) with ozone-oxygen mixture at concentrations that are calculated depending on the patient’s weight (ranging 1–5 µg/kg b.w.). Its administration takes about 20–30 min. Unlike MAH, the O_3_SS has been used as complementary therapy in viral diseases such as Epstein Barr, Cytomegalovirus, Papillomavirus, HIV, Herpes zoster, Herpes simplex, etc. Since the saline solution is a plasma expander, O_3_SS represents a greater amount of blood being treated than MAH and therefore, theoretically, the number of sessions could be reduced.

Korolev, B.A., Boyarinov, G.A. and Sokolov, V.V. [54,55] showed that when an O_3_SS was used during cardiopulmonary bypass, the cells of the patient’s organs use more glucose compared to basal levels. Therefore, it is concluded that the therapeutic effects of ozonated physiological solutions, is determined by the dissolved O_2_/O_3_ mixture, free radicals, hydrogen peroxide and hexagonal aqueous structures formed during the bubbling of aqueous NaCl solutions with a mixture of O_2_/O_3_ gas. 

## 3. Ozone Therapy as Redox Modulator

During a systemic application of O_2_/O_3_ (mainly MAH, O_3_SS, vaginal and rectal insufflation), part of the O_3_ is removed by the antioxidants of the medium. Further reaction of O_3_ with biomolecules generates aldehyde (e.g., 4-hydroxynonenal (4-HNE)) and peroxide (H_2_O_2_ and organic peroxides). These byproducts of the reaction act as secondary messengers and induce a further adaptive hormetic responses [56,57,58]. Ozone at a therapeutic dose “only acts” as a modulator or pro-drug and, by inducing secondary messengers, will enhance subsequent adaptive responses [21]. 

Mediators such as 4-HNE and H_2_O_2_ are among the most relevant secondary messengers producing beneficial effects during medical applications, they induce a gradual oxidative stimulus, that produces the synthesis of endogenous antioxidants such as SOD, CAT and GPx [18,59]. This fact implies that O_2_/O_3_ is a paradoxical pro-oxidant therapy that invokes an endogenous antioxidant response. Moreover, low quantities of H_2_O_2_ formed as consequence of O_2_/O_3_ have a key role in the molecular mechanism. H_2_O_2_ is crucial and a common activator of the modulation of NF-κB and Nrf2 pathways [60,61,62]. In addition, 4-HNE also sends a signal of transient oxidative stress and its effects depend on concentration as well as cell/tissue origin. This pathway can activate the synthesis of several substances such as: γ-glutamyl transferase, γ-glutamyl transpeptidase, HSP-70, HO-1, and antioxidant enzymes such as SOD, GPx, CAT and glucose-6-phosphate dehydrogenase [21]. In addition, these pluripotent effects of 4-HNE can be explained by its concentration-dependent interactions with the cytokine networks and complex cellular antioxidant systems also showing cell and tissue specificities [59,63].

**Figure 1:**
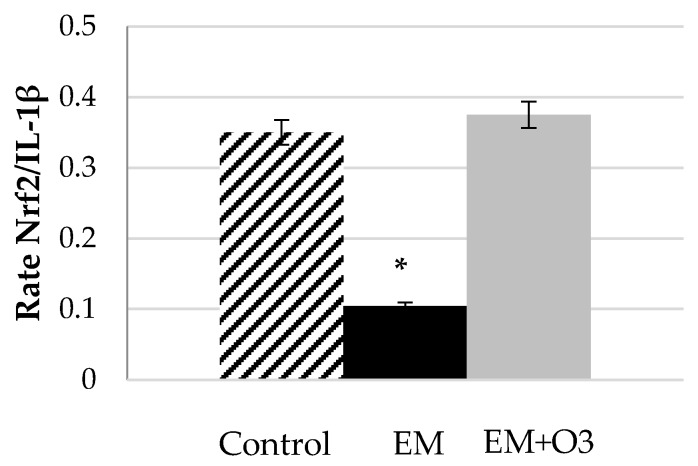
Rate Nrf2/IL-1β as biomarkers of balance Nrf2/NF-κB pathway activation after and before O_2_/O_3_ treatment. Control group, healthy volunteers; EM, Multiple sclerosis relapsing-remitting patients with not exacerbation episodes of the disease; EM + O3, Multiple sclerosis patients after O_2_/O_3_ treatment by rectal insufflation for 30 days (three times per week during a month at 20 μg/mL). Data was taken and proceeded from Delgado et al., 2017 [19]. Values represent mean ± S.E.M. of three independent experiments (* *p* < 0.05).

Experimental results demonstrated that ozone at therapeutic dosages ex vivo or in vivo can activate Nrf2 [20,32] that involve an indirect modulation (inhibition) of the NF-κB pathway. In addition, Nrf2 suppresses NF-κB activity by eliminating ROS, which may cause NF-κB activation via antioxidative protein induction, such as HO-1 and NQO1. Moreover, Nrf2 suppresses NF-κB activity through some protein–protein interactions, and also suppresses an inflammatory cytokine gene expression through binding to their gene promoter directly [64]. NF-κB pathway activates the release of pro-inflammatory cytokines like: TNFα, IFNγ, IL1β, IL6, IL8, as well as pro-inflammatory genes likecyclooxygenase-2 (COX-2) and inducible nitric oxide synthase (iNOS) [65]. It is well known that the regulation of both pathways, NF-κB and Nrf2, involved a crosstalk to bring a coordinated inflammatory response [66,67]. The modulation of the inflammatory response by ozone was evident in a clinical trial l on patients with multiple sclerosis (ME) treated with O_2_/O_3_ by rectal insufflation for 30 days [19]. Taking the original data of this trail and recalculating the values in terms of the ratio Nrf2 phosphorylation (as expression of activation of Nrf2 pathway) and IL-1β (as marked of the NF-κB pathway) this modulation became evident (Figure 1). In patients with ME without treatment, the balance Nrf2/NF-κB favor the inflammatory process, and O_2_/O_3_ restores the balance of those pathways.

An individual analysis of the variable assayed in this study shown a significant (*p* < 0.05) increase in Nrf2 values in ozone treated patients compared to control group (0.93 vs. 0.75 densitometry unit, respectively), that restore the significant downregulated values of Nrf2 at basal level in ME patients (0.56 densitometric unit). The increment in Nrf2 was in line with the significant reduction in 61% of the level of the pro inflammatory cytokine IL-1β in ME-treated patients compared with basal levels, even IL-1β levels in ME remain 94% higher than values in normal subjects.

An imbalance between Nrf2/NF-κB has been proposed in other diseases like diabetic neuropathy [68] in which ozone experimentally demonstrated its efficacy equilibrating this disruption [62]. Similar trends were found in viral diseases, NF-κB pathways can support influenza A virus infection and promote pneumonia. Through the Activation of the Nrf2 signaling some drug as emodin can increased the survival rate, reduce lung edema, pulmonary viral titer and inflammatory cytokines, and improve lung histopathological changes [69]. In addition, it has been shown that the rabbit hemorrhagic disease virus (that causes lethal fulminant hepatitis in rabbits) has a pathological mechanism that involves is the repression of Nrf2 pathway [70].

## 4. Ozone Therapy and Cytoprotection

Antioxidants are important for the maintenance of cellular integrity and cytoptotection. Modulating the balance Nrf2/NF-κB, O_2_/O_3_ not only increases the endogenous antioxidant system but also modulates the expression of pro-inflammatory cytokines and has an impact in cytoprotection. COVID-19 infects the upper and lower respiratory tracts and causes mild to highly acute respiratory syndrome with consequent over-expression of pro-inflammatory cytokines, including interleukin IL-1β and IL-6. Activation of toll-like receptors by SARS Cov-2 RNA lead to the release of pro-IL-1β which is cleaved by caspase-1, followed by inflammatory activation and production of active mature IL-1β which is a mediator of lung inflammation, fever and fibrosis [71]. However, suppression but not depletion of the pro-inflammatory IL-1 family and IL-6 have been shown to have a therapeutic effect in many inflammatory diseases, including viral infections for instance, Mice lacking IL-1 signaling expression, elevated viral replication of coronavirus [72]. In addition, IL-6-deficient mice infected with influenza virus exhibited a higher lethality, more body weight loss and had higher fibroblast accumulation and lower extracellular matrix (ECM) turnover in the lungs than their wild-type counterparts [73]. The inflammasome, a cytosolic protein complex that mediates the processing and secretion of pro-inflammatory cytokines, is one of the first responders during viral infection. The cytokines secreted, following inflammasome activation, regulate cells of both the innate and adaptive immune system, guiding the subsequent immune responses. Therefore, not suppressive but a modulator of cytokines may impact efficiently in vital cytotoxicity. A representative data about the downregulated of cytokines IL-1β, IL-6, IL-8 and TNF-α are shown in Table 1. 

**Figure 2:**
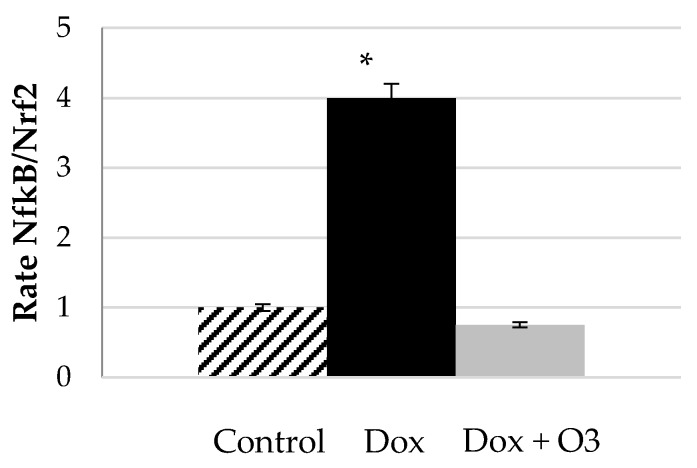
Rate of the fold change values of NF-κB/Nrf2 as index of balance NF-κB/Nrf2 pathway activation with and without O_2_/O_3_ treatment. Control, cardiomyocytes culture; Dox, cells plus doxorubicin (100 nM); Dox + O3, cell treated with doxorubicin (100 nM) and ozone 40 μg/mL. Data were taken and proceeded from Simonetti et al., 2019 [44]. Values represented a mean ± S.E.M. of three independent experiments (* *p* < 0.01).

The expression of cytokine responses to a previous signal is closely connected with the action of nuclear factors. An in vitro experiment, conducted in cardiomyocytes and skin fibroblasts, analysed the role of ozone at the level of Nrf2 and NF-κB induced by doxorubicin [44]. The authors analysed the individual role of different doses of ozone in this model. A re-analysis of this data, calculating the rate NF-κB/Nrf2, showed the evident downregulation of the effect of ozone but not suppression (Figure 2). The same trend was observed also in skin fibroblasts cultures treated with doxorubicin and O_2_/O_3_.

An analysis of the individual values showed that the restoration of the equilibrium NF-κB/Nrf2 was reached essentially by the preservation of the level of Nrf2 in ozone-treated cells (0.8 vs. 0.9 fold of chance, in control group, respectively), compared with the depleted values of Nrf2 observed in doxorubicin-treated cells (0.5, fold of chance, with respect to control cell culture). This maintenance of Nrf2 levels avoids the 100% increment in NF-κB that takes place during the doxorubicin treatment. The experiment showed that the intervention with ozone, preserve Nrf2 essential facts to avoid and up-regulate NF-κB.

The hormetic response, oxidative preconditioning or the adaptation to the chronic oxidative stress, during OT, has been now demonstrated experimentally [78]. The concept of doses is very remarkable in OT, the administration route and clinical protocols are also important. At higher doses the described effect of ozone could drastically change. High doses of ozone induce the gene transcription of the pro-inflammatory cytokine, its receptor, and inflammatory proteins. At the same time, they invoke a negative regulation of type 1 Interferon and the response to viral infections pathways [79].

The recommended systemic administration routes are: O_3_SS, MAH and Extracorporeal Blood Oxygenation-Ozonation (EBOO). Clinical protocols should comply with the standard doses and procedures defined in the Madrid Declaration of OT [16]. At least three clinical trials using major autohemotherapy are in progress in China and more clinical trials are needed to confirm the efficacy of OT as complementary therapy in the treatment of COVID-19 diseases. It is a complementary therapy because, while the infected patient is treated with allopathic medicine, at the same time the patient will also receive the complementary proposed treatment. It should be noted that, even if ozone has no effect on the virus infection, the demonstrated modulation of oxidative stress and inflammatory cytokines by ozone therapy could offer a relevant and beneficial clinical effect. Additionally, a small impact in the requirements of inpatients days, especially on intensive care units, could lead to a high benefit in the current critical situation that many countries are suffering.

## 5. Conclusions

Systemic OT can be potentially useful in SARS-CoV-2. The rationale and mechanism of action have already been proven clinically with other viral infections and have been shown to be highly effective in research studies. The mechanisms of action involved are the modulation of the NF-κB/Nrf2 pathway and IL-6/IL-1β expression. The modulation of these pathways by OT have an impact in the cytoptotection and blockage of viral replication. Future clinical trials are needed to prove the complementary use of OT in COVID-19.

## Figures and Tables

**Table 1:** Effect of ozone therapy as modulator of pro-inflammatory cytokines.

Type of Study	Model/Target Organ	Respond in Term of Cytokines	Main Result/Reference
In vivo (rats)	Sepsis/Spleen and liver	IL-1β↓/TNF-α↓	Increased survival rate in 33% [74].
In vivo (rats)	Chronic Radiculitis/Spinal	TNF-α↓/IL-1β↓/IL-6↓	Alleviated mechanical allodynia and attenuated radiculitis [75].
In vitro	Odontoblastic cell line, Sepsis induced by LPS	IL-6↓, TNF-α↓	Inhibition of inflammatory response [76].
In vitro	Human skin fibroblast cells and human fetal cardiomyocytes, Damage induced by doxorubicin	IL-1β↓, IL-8↓, IL-6↓, TNF-α↓	Significantly decreased the cytotoxicity [44].
In vivo (rats)	Ischemia-reperfusion injury/lung	IL-1β↓	Lung cytoprotection [77].

Legend: ↓, downregulation; LPS, lipopolysaccharides.

## Abbreviations

4-HNE	4-hydroxynonenal
ACE2	angiotensin-converting enzyme 2
CAT	catalase
AEMPS	Spanish Drug Agency and Health Products
COX-2	cyclooxygenase-2
CT	computed tomography
ECM	extracellular matrix
EBOO	Extracorporeal Blood Oxygenation-Ozonation
FDA	Food and Drug Administration
GPx	glutathione peroxidase
HSP	heat shock proteins
HO-1	heme oxygenase-1
H_2_O_2_	hydrogen peroxide
IL	interleukin
MAH	mayor autohemotherapy
ME	multiple sclerosis
MSCs	Derived Mesenchymal Stem Cells
NF-κB	nuclear factor kappa B
Nrf2	nuclear factor erythroid 2-related factor
S.E.M.	standard error of the mean
O_2_	oxygen
O_3_	ozone
OT	ozone therapy
O_3_SS	Ozonized Saline Solution
SARS-CoV-2	severe acute respiratory syndrome coronavirus 2
SOD	superoxide dismutase
TLR	Toll Like Receptor
TNF-α	tumor necrosis factor-alpha
UC	Umbilical Cord
WHO	world health organization
